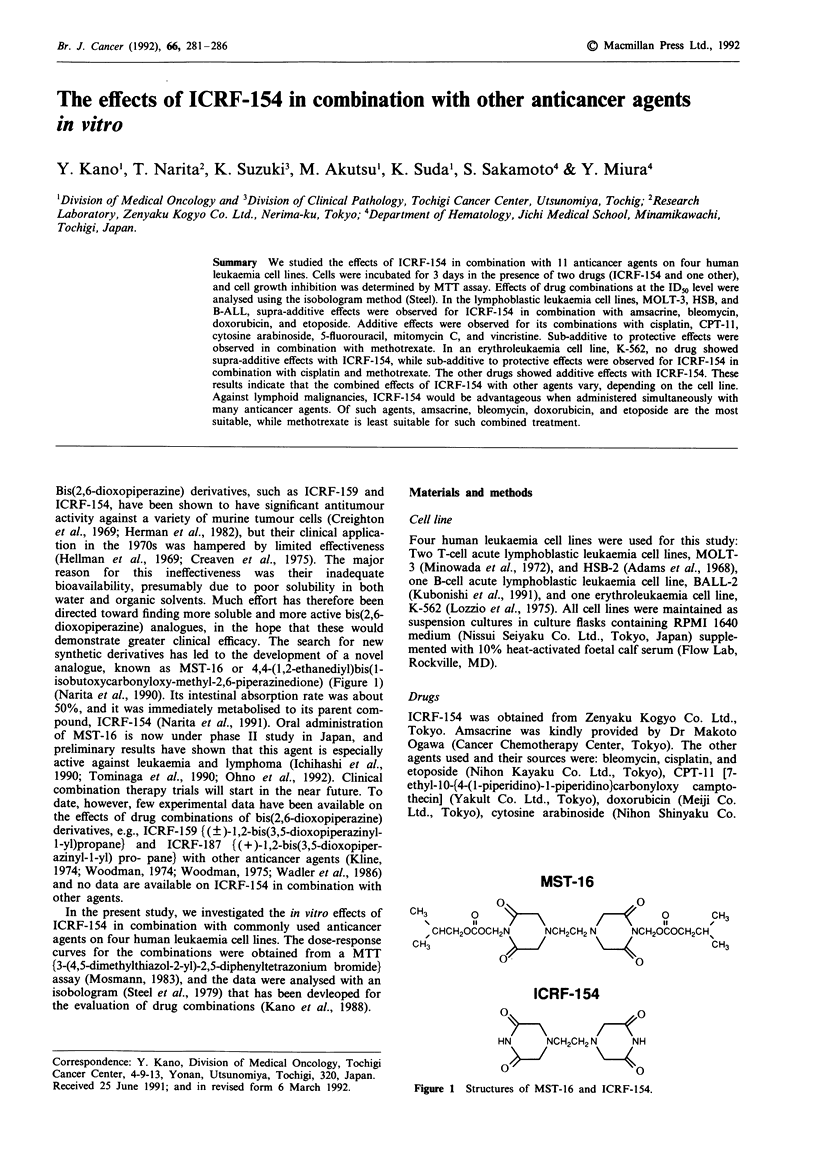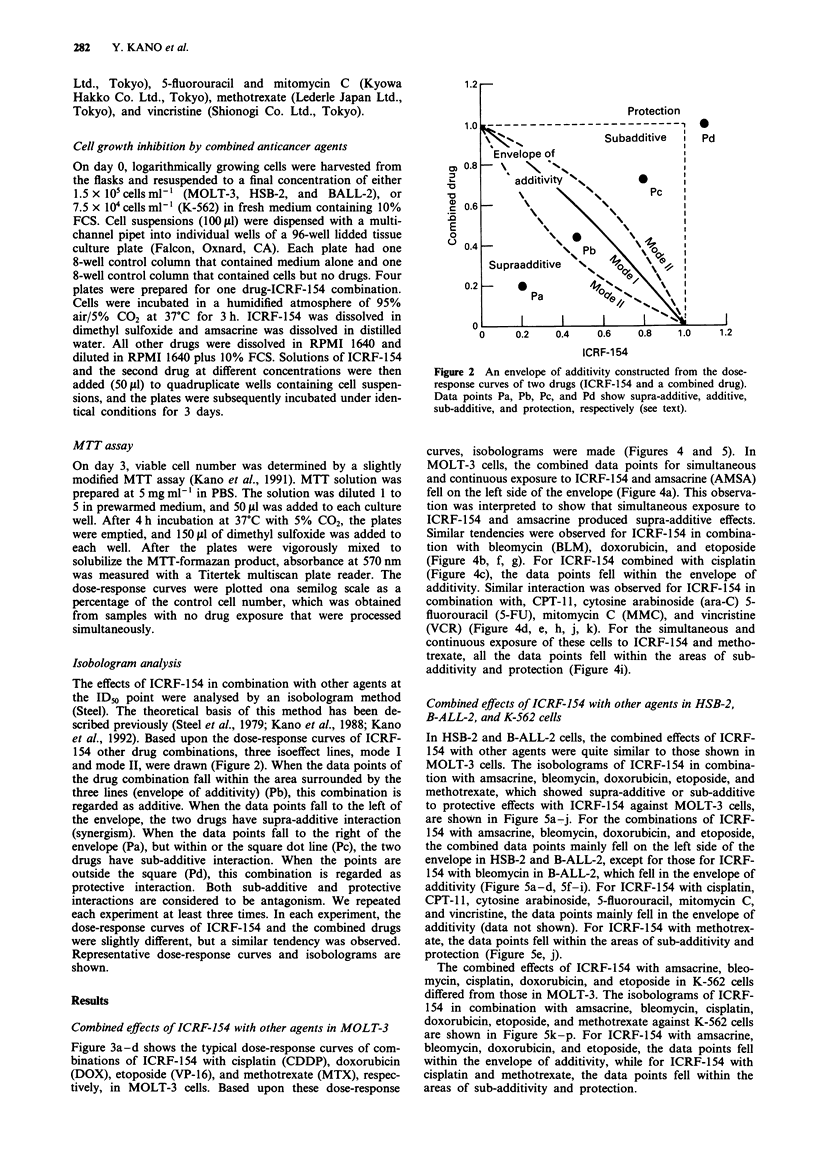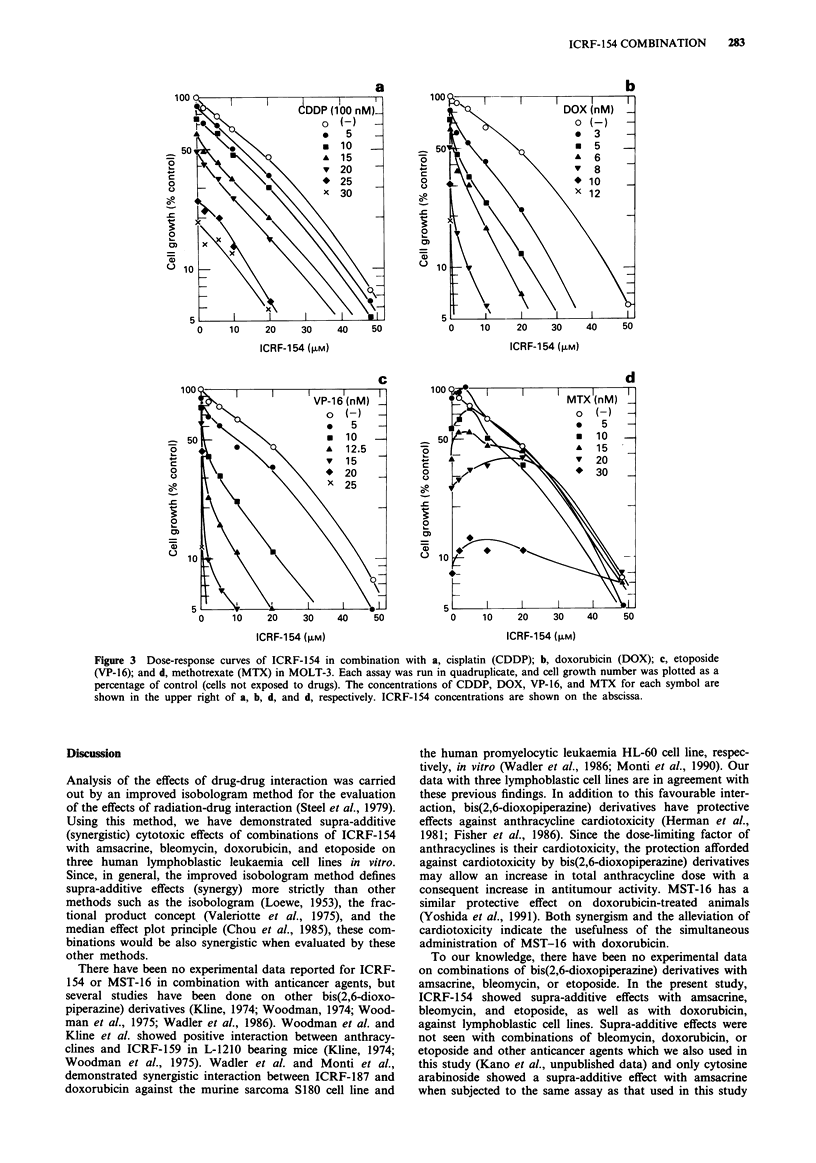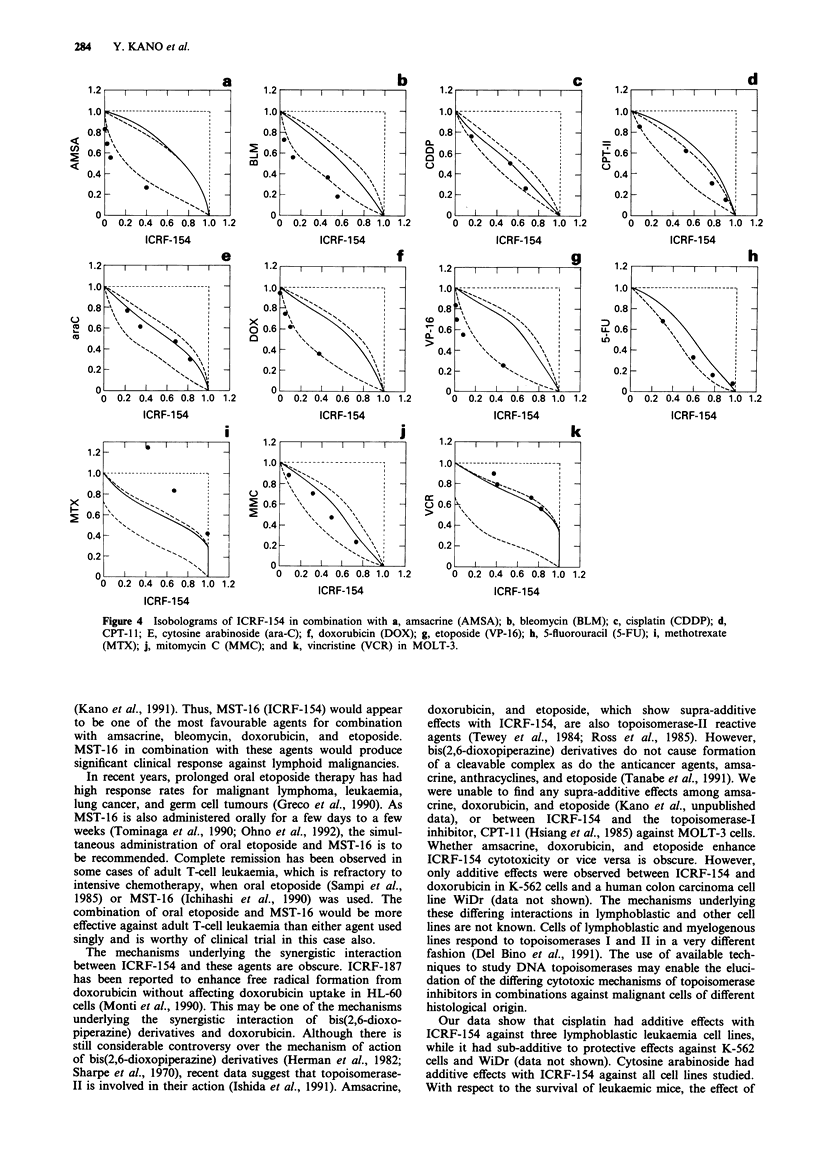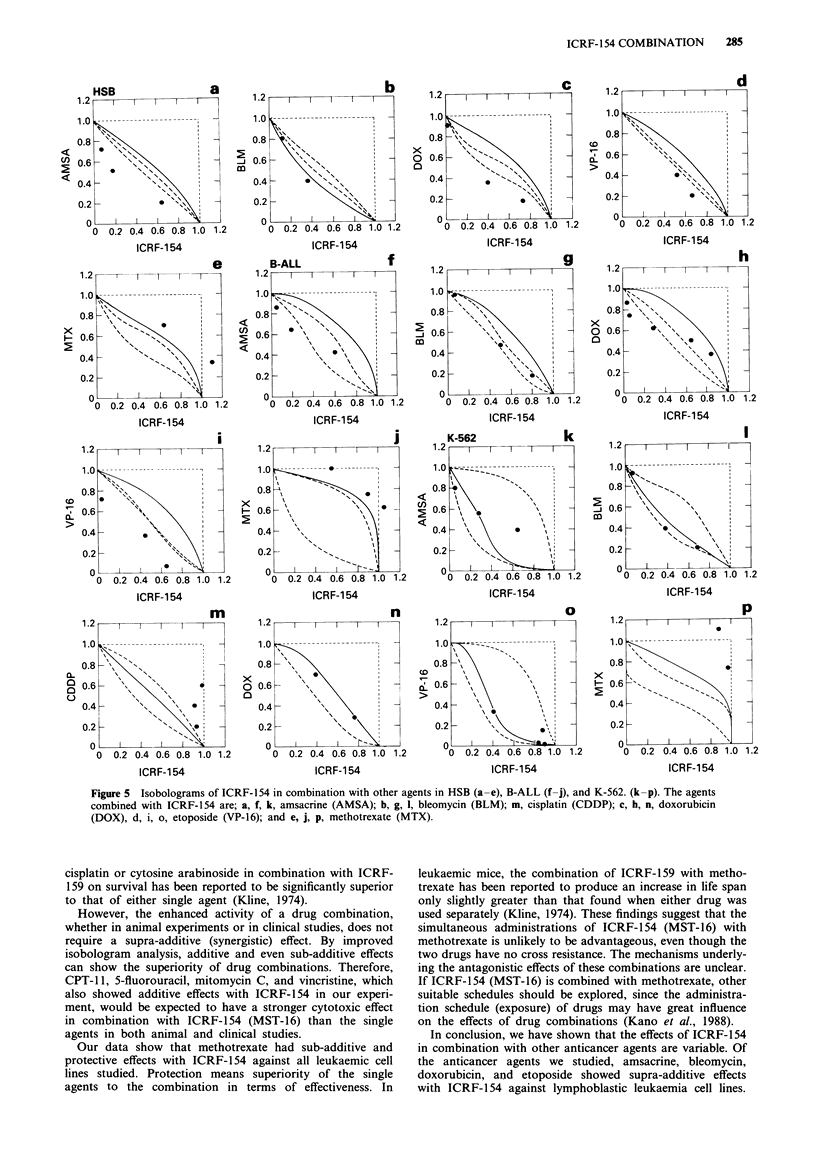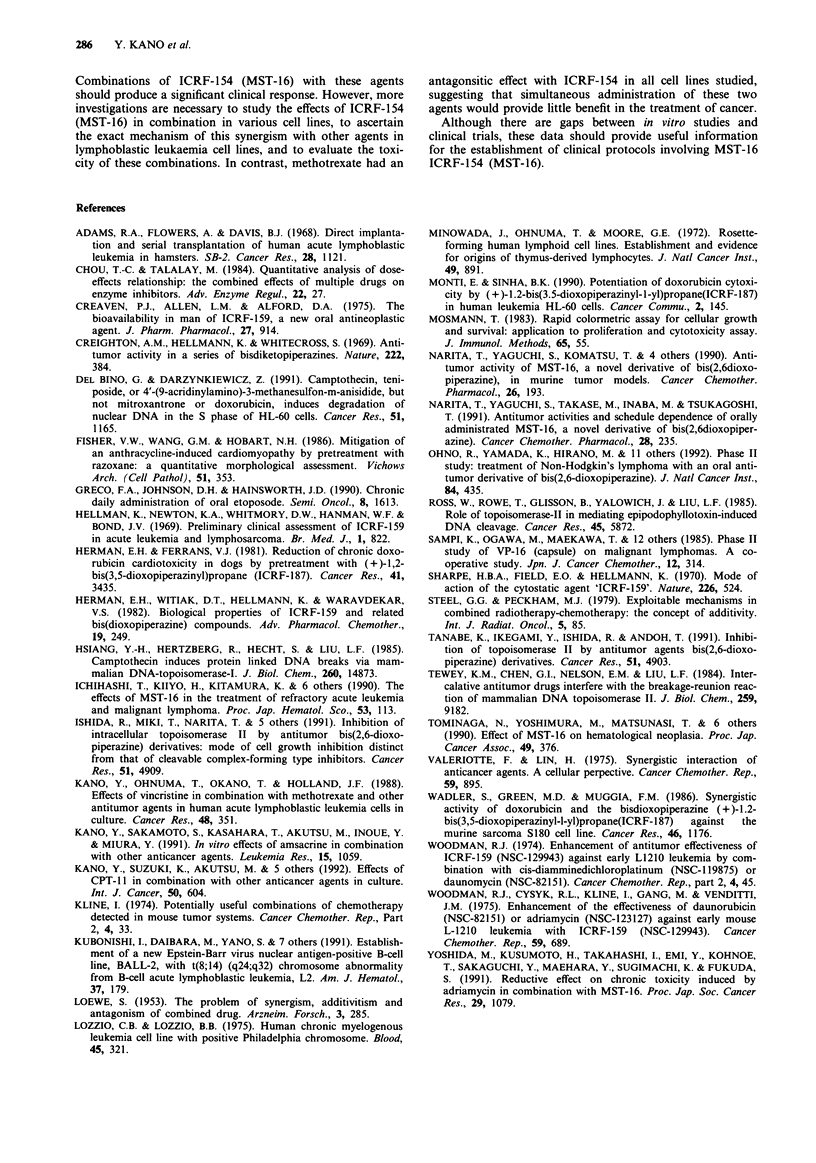# The effects of ICRF-154 in combination with other anticancer agents in vitro.

**DOI:** 10.1038/bjc.1992.257

**Published:** 1992-08

**Authors:** Y. Kano, T. Narita, K. Suzuki, M. Akutsu, K. Suda, S. Sakamoto, Y. Miura

**Affiliations:** Division of Medical Oncology, Tochigi Cancer Center, Japan.

## Abstract

We studied the effects of ICRF-154 in combination with 11 anticancer agents on four human leukaemia cell lines. Cells were incubated for 3 days in the presence of two drugs (ICRF-154 and one other), and cell growth inhibition was determined by MTT assay. Effects of drug combinations at the ID50 level were analysed using the isobologram method (Steel). In the lymphoblastic leukaemia cell lines, MOLT-3, HSB, and B-ALL, supra-additive effects were observed for ICRF-154 in combination with amsacrine, bleomycin, doxorubicin, and etoposide. Additive effects were observed for its combinations with cisplatin, CPT-11, cytosine arabinoside, 5-fluorouracil, mitomycin C, and vincristine. Sub-additive to protective effects were observed in combination with methotrexate. In an erythroleukaemia cell line, K-562, no drug showed supra-additive effects with ICRF-154, while sub-additive to protective effects were observed for ICRF-154 in combination with cisplatin and methotrexate. The other drugs showed additive effects with ICRF-154. These results indicate that the combined effects of ICRF-154 with other agents vary, depending on the cell line. Against lymphoid malignancies, ICRF-154 would be advantageous when administered simultaneously with many anticancer agents. Of such agents, amsacrine, bleomycin, doxorubicin, and etoposide are the most suitable, while methotrexate is least suitable for such combined treatment.


Br. J. Cancer (1992), 66, 281-286 © Macmillan Press Ltd., 1992

### The effects of ICRF-154 in combination with other anticancer agents *in vitro*

Y. Kano[1], T. Narita[2], K. Suzuki[3], M. Akutsu[1], K. Suda[1], S. Sakamoto[4] & Y. Miura[4]

[1]*Division of Medical Oncology and* [3]*Division of Clinical Pathology, Tochigi Cancer Center, Utsunomiya, Tochig;* [2]*Research Laboratory, Zenyaku Kogyo Co. Ltd., Nerima-ku, Tokyo;* [4]*Department of Hematology, Jichi Medical School, Minamikawachi, Tochigi, Japan.*

**Summary** We studied the effects of ICRF-154 in combination with 11 anticancer agents on four human leukaemia cell lines. Cells were incubated for 3 days in the presence of two drugs (ICRF-154 and one other), and cell growth inhibition was determined by MTT assay. Effects of drug combinations at the $ID_{50}$ level were analysed using the isobologram method (Steel). In the lymphoblastic leukaemia cell lines, MOLT-3, HSB, and B-ALL, supra-additive effects were observed for ICRF-154 in combination with amsacrine, bleomycin, doxorubicin, and etoposide. Additive effects were observed for its combinations with cisplatin, CPT-11, cytosine arabinoside, 5-fluorouracil, mitomycin C, and vincristine. Sub-additive to protective effects were observed in combination with methotrexate. In an erythroleukaemia cell line, K-562, no drug showed supra-additive effects with ICRF-154, while sub-additive to protective effects were observed for ICRF-154 in combination with cisplatin and methotrexate. The other drugs showed additive effects with ICRF-154. These results indicate that the combined effects of ICRF-154 with other agents vary, depending on the cell line. Against lymphoid malignancies, ICRF-154 would be advantageous when administered simultaneously with many anticancer agents. Of such agents, amsacrine, bleomycin, doxorubicin, and etoposide are the most suitable, while methotrexate is least suitable for such combined treatment.

Bis(2,6-dioxopiperazine) derivatives, such as ICRF-159 and ICRF-154, have been shown to have significant antitumour activity against a variety of murine tumour cells (Creighton *et al.*, 1969; Herman *et al.*, 1982), but their clinical application in the 1970s was hampered by limited effectiveness (Hellman *et al.*, 1969; Creaven *et al.*, 1975). The major reason for this ineffectiveness was their inadequate bioavailability, presumably due to poor solubility in both water and organic solvents. Much effort has therefore been directed toward finding more soluble and more active bis(2,6-dioxopiperazine) analogues, in the hope that these would demonstrate greater clinical efficacy. The search for new synthetic derivatives has led to the development of a novel analogue, known as MST-16 or 4,4-(1,2-ethanediyl)bis(1-isobutoxycarbonyloxy-methyl-2,6-piperazinedione) (Figure 1) (Narita *et al.*, 1990). Its intestinal absorption rate was about 50%, and it was immediately metabolised to its parent compound, ICRF-154 (Narita *et al.*, 1991). Oral administration of MST-16 is now under phase II study in Japan, and preliminary results have shown that this agent is especially active against leukaemia and lymphoma (Ichihashi *et al.*, 1990; Tominaga *et al.*, 1990; Ohno *et al.*, 1992). Clinical combination therapy trials will start in the near future. To date, however, few experimental data have been available on the effects of drug combinations of bis(2,6-dioxopiperazine) derivatives, e.g., ICRF-159 {(±)-1,2-bis(3,5-dioxopiperazinyl-1-yl)propane} and ICRF-187 {(+)-1,2-bis(3,5-dioxopiperazinyl-1-yl) pro- pane} with other anticancer agents (Kline, 1974; Woodman, 1974; Woodman, 1975; Wadler *et al.*, 1986) and no data are available on ICRF-154 in combination with other agents.

In the present study, we investigated the *in vitro* effects of ICRF-154 in combination with commonly used anticancer agents on four human leukaemia cell lines. The dose-response curves for the combinations were obtained from a MTT {3-(4,5-dimethylthiazol-2-yl)-2,5-diphenyltetrazonium bromide} assay (Mosmann, 1983), and the data were analysed with an isobologram (Steel *et al.*, 1979) that has been devleoped for the evaluation of drug combinations (Kano *et al.*, 1988).

Correspondence: Y. Kano, Division of Medical Oncology, Tochigi Cancer Center, 4-9-13, Yonan, Utsunomiya, Tochigi, 320, Japan.
Received 25 June 1991; and in revised form 6 March 1992.

### Materials and methods

### Cell line

Four human leukaemia cell lines were used for this study: Two T-cell acute lymphoblastic leukaemia cell lines, MOLT-3 (Minowada *et al.*, 1972), and HSB-2 (Adams *et al.*, 1968), one B-cell acute lymphoblastic leukaemia cell line, BALL-2 (Kubonishi *et al.*, 1991), and one erythroleukaemia cell line, K-562 (Lozzio *et al.*, 1975). All cell lines were maintained as suspension cultures in culture flasks containing RPMI 1640 medium (Nissui Seiyaku Co. Ltd., Tokyo, Japan) supplemented with 10% heat-activated foetal calf serum (Flow Lab, Rockville, MD).

### Drugs

ICRF-154 was obtained from Zenyaku Kogyo Co. Ltd., Tokyo. Amsacrine was kindly provided by Dr Makoto Ogawa (Cancer Chemotherapy Center, Tokyo). The other agents used and their sources were: bleomycin, cisplatin, and etoposide (Nihon Kayaku Co. Ltd., Tokyo), CPT-11 [7-ethyl-10-{4-(1-piperidino)-1-piperidino}carbonyloxy camptothecin] (Yakult Co. Ltd., Tokyo), doxorubicin (Meiji Co. Ltd., Tokyo), cytosine arabinoside (Nihon Shinyaku Co.

**Figure 1** Structures of MST-16 and ICRF-154.

Ltd., Tokyo), 5-fluorouracil and mitomycin C (Kyowa Hakko Co. Ltd., Tokyo), methotrexate (Lederle Japan Ltd., Tokyo), and vincristine (Shionogi Co. Ltd., Tokyo).

*Cell growth inhibition by combined anticancer agents*

On day 0, logarithmically growing cells were harvested from the flasks and resuspended to a final concentration of either $1.5 \times 10^5$ cells $ml^{-1}$ (MOLT-3, HSB-2, and BALL-2), or $7.5 \times 10^4$ cells $ml^{-1}$ (K-562) in fresh medium containing 10% FCS. Cell suspensions (100 µl) were dispensed with a multichannel pipet into individual wells of a 96-well lidded tissue culture plate (Falcon, Oxnard, CA). Each plate had one 8-well control column that contained medium alone and one 8-well control column that contained cells but no drugs. Four plates were prepared for one drug-ICRF-154 combination. Cells were incubated in a humidified atmosphere of 95% air/5% $CO_2$ at 37°C for 3 h. ICRF-154 was dissolved in dimethyl sulfoxide and amsacrine was dissolved in distilled water. All other drugs were dissolved in RPMI 1640 and diluted in RPMI 1640 plus 10% FCS. Solutions of ICRF-154 and the second drug at different concentrations were then added (50 µl) to quadruplicate wells containing cell suspensions, and the plates were subsequently incubated under identical conditions for 3 days.

*MTT assay*

On day 3, viable cell number was determined by a slightly modified MTT assay (Kano *et al.*, 1991). MTT solution was prepared at 5 mg $ml^{-1}$ in PBS. The solution was diluted 1 to 5 in prewarmed medium, and 50 µl was added to each culture well. After 4 h incubation at 37°C with 5% $CO_2$, the plates were emptied, and 150 µl of dimethyl sulfoxide was added to each well. After the plates were vigorously mixed to solubilize the MTT-formazan product, absorbance at 570 nm was measured with a Titertek multiscan plate reader. The dose-response curves were plotted ona semilog scale as a percentage of the control cell number, which was obtained from samples with no drug exposure that were processed simultaneously.

*Isobologram analysis*

The effects of ICRF-154 in combination with other agents at the $ID_{50}$ point were analysed by an isobologram method (Steel). The theoretical basis of this method has been described previously (Steel *et al.*, 1979; Kano *et al.*, 1988; Kano *et al.*, 1992). Based upon the dose-response curves of ICRF-154 other drug combinations, three isoeffect lines, mode I and mode II, were drawn (Figure 2). When the data points of the drug combination fall within the area surrounded by the three lines (envelope of additivity) (Pb), this combination is regarded as additive. When the data points fall to the left of the envelope, the two drugs have supra-additive interaction (synergism). When the data points fall to the right of the envelope (Pa), but within or the square dot line (Pc), the two drugs have sub-additive interaction. When the points are outside the square (Pd), this combination is regarded as protective interaction. Both sub-additive and protective interactions are considered to be antagonism. We repeated each experiment at least three times. In each experiment, the dose-response curves of ICRF-154 and the combined drugs were slightly different, but a similar tendency was observed. Representative dose-response curves and isobolograms are shown.

### Results

*Combined effects of ICRF-154 with other agents in MOLT-3*

Figure 3a–d shows the typical dose-response curves of combinations of ICRF-154 with cisplatin (CDDP), doxorubicin (DOX), etoposide (VP-16), and methotrexate (MTX), respectively, in MOLT-3 cells. Based upon these dose-response curves, isobolograms were made (Figures 4 and 5). In MOLT-3 cells, the combined data points for simultaneous and continuous exposure to ICRF-154 and amsacrine (AMSA) fell on the left side of the envelope (Figure 4a). This observation was interpreted to show that simultaneous exposure to ICRF-154 and amsacrine produced supra-additive effects. Similar tendencies were observed for ICRF-154 in combination with bleomycin (BLM), doxorubicin, and etoposide (Figure 4b, f, g). For ICRF-154 combined with cisplatin (Figure 4c), the data points fell within the envelope of additivity. Similar interaction was observed for ICRF-154 in combination with, CPT-11, cytosine arabinoside (ara-C) 5-fluorouracil (5-FU), mitomycin C (MMC), and vincristine (VCR) (Figure 4d, e, h, j, k). For the simultaneous and continuous exposure of these cells to ICRF-154 and methotrexate, all the data points fell within the areas of sub-additivity and protection (Figure 4i).

**Figure 2** An envelope of additivity constructed from the dose-response curves of two drugs (ICRF-154 and a combined drug). Data points Pa, Pb, Pc, and Pd show supra-additive, additive, sub-additive, and protection, respectively (see text).

*Combined effects of ICRF-154 with other agents in HSB-2, B-ALL-2, and K-562 cells*

In HSB-2 and B-ALL-2 cells, the combined effects of ICRF-154 with other agents were quite similar to those shown in MOLT-3 cells. The isobolograms of ICRF-154 in combination with amsacrine, bleomycin, doxorubicin, etoposide, and methotrexate, which showed supra-additive or sub-additive to protective effects with ICRF-154 against MOLT-3 cells, are shown in Figure 5a–j. For the combinations of ICRF-154 with amsacrine, bleomycin, doxorubicin, and etoposide, the combined data points mainly fell on the left side of the envelope in HSB-2 and B-ALL-2, except for those for ICRF-154 with bleomycin in B-ALL-2, which fell in the envelope of additivity (Figure 5a–d, 5f–i). For ICRF-154 with cisplatin, CPT-11, cytosine arabinoside, 5-fluorouracil, mitomycin C, and vincristine, the data points mainly fell in the envelope of additivity (data not shown). For ICRF-154 with methotrexate, the data points fell within the areas of sub-additivity and protection (Figure 5e, j).

The combined effects of ICRF-154 with amsacrine, bleomycin, cisplatin, doxorubicin, and etoposide in K-562 cells differed from those in MOLT-3. The isobolograms of ICRF-154 in combination with amsacrine, bleomycin, cisplatin, doxorubicin, etoposide, and methotrexate against K-562 cells are shown in Figure 5k–p. For ICRF-154 with amsacrine, bleomycin, doxorubicin, and etoposide, the data points fell within the envelope of additivity, while for ICRF-154 with cisplatin and methotrexate, the data points fell within the areas of sub-additivity and protection.

**Figure 3** Dose-response curves of ICRF-154 in combination with **a**, cisplatin (CDDP); **b**, doxorubicin (DOX); **c**, etoposide (VP-16); and **d**, methotrexate (MTX) in MOLT-3. Each assay was run in quadruplicate, and cell growth number was plotted as a percentage of control (cells not exposed to drugs). The concentrations of CDDP, DOX, VP-16, and MTX for each symbol are shown in the upper right of **a**, **b**, **d**, and **d**, respectively. ICRF-154 concentrations are shown on the abscissa.

### Discussion

Analysis of the effects of drug-drug interaction was carried out by an improved isobologram method for the evaluation of the effects of radiation-drug interaction (Steel *et al.*, 1979). Using this method, we have demonstrated supra-additive (synergistic) cytotoxic effects of combinations of ICRF-154 with amsacrine, bleomycin, doxorubicin, and etoposide on three human lymphoblastic leukaemia cell lines *in vitro*. Since, in general, the improved isobologram method defines supra-additive effects (synergy) more strictly than other methods such as the isobologram (Loewe, 1953), the fractional product concept (Valeriotte *et al.*, 1975), and the median effect plot principle (Chou *et al.*, 1985), these combinations would be also synergistic when evaluated by these other methods.

There have been no experimental data reported for ICRF-154 or MST-16 in combination with anticancer agents, but several studies have been done on other bis(2,6-dioxopiperazine) derivatives (Kline, 1974; Woodman, 1974; Woodman *et al.*, 1975; Wadler *et al.*, 1986). Woodman *et al.* and Kline *et al.* showed positive interaction between anthracyclines and ICRF-159 in L-1210 bearing mice (Kline, 1974; Woodman *et al.*, 1975). Wadler *et al.* and Monti *et al.*, demonstrated synergistic interaction between ICRF-187 and doxorubicin against the murine sarcoma S180 cell line and the human promyelocytic leukaemia HL-60 cell line, respectively, *in vitro* (Wadler *et al.*, 1986; Monti *et al.*, 1990). Our data with three lymphoblastic cell lines are in agreement with these previous findings. In addition to this favourable interaction, bis(2,6-dioxopiperazine) derivatives have protective effects against anthracycline cardiotoxicity (Herman *et al.*, 1981; Fisher *et al.*, 1986). Since the dose-limiting factor of anthracyclines is their cardiotoxicity, the protection afforded against cardiotoxicity by bis(2,6-dioxopiperazine) derivatives may allow an increase in total anthracycline dose with a consequent increase in antitumour activity. MST-16 has a similar protective effect on doxorubicin-treated animals (Yoshida *et al.*, 1991). Both synergism and the alleviation of cardiotoxicity indicate the usefulness of the simultaneous administration of MST–16 with doxorubicin.

To our knowledge, there have been no experimental data on combinations of bis(2,6-dioxopiperazine) derivatives with amsacrine, bleomycin, or etoposide. In the present study, ICRF-154 showed supra-additive effects with amsacrine, bleomycin, and etoposide, as well as with doxorubicin, against lymphoblastic cell lines. Supra-additive effects were not seen with combinations of bleomycin, doxorubicin, or etoposide and other anticancer agents which we also used in this study (Kano *et al.*, unpublished data) and only cytosine arabinoside showed a supra-additive effect with amsacrine when subjected to the same assay as that used in this study

**Figure 4** Isobolograms of ICRF-154 in combination with **a**, amsacrine (AMSA); **b**, bleomycin (BLM); **c**, cisplatin (CDDP); **d**, CPT-11; E, cytosine arabinoside (ara-C); **f**, doxorubicin (DOX); **g**, etoposide (VP-16); **h**, 5-fluorouracil (5-FU); **i**, methotrexate (MTX); **j**, mitomycin C (MMC); and **k**, vincristine (VCR) in MOLT-3.

(Kano *et al.*, 1991). Thus, MST-16 (ICRF-154) would appear to be one of the most favourable agents for combination with amsacrine, bleomycin, doxorubicin, and etoposide. MST-16 in combination with these agents would produce significant clinical response against lymphoid malignancies.

In recent years, prolonged oral etoposide therapy has had high response rates for malignant lymphoma, leukaemia, lung cancer, and germ cell tumours (Greco *et al.*, 1990). As MST-16 is also administered orally for a few days to a few weeks (Tominaga *et al.*, 1990; Ohno *et al.*, 1992), the simultaneous administration of oral etoposide and MST-16 is to be recommended. Complete remission has been observed in some cases of adult T-cell leukaemia, which is refractory to intensive chemotherapy, when oral etoposide (Sampi *et al.*, 1985) or MST-16 (Ichihashi *et al.*, 1990) was used. The combination of oral etoposide and MST-16 would be more effective against adult T-cell leukaemia than either agent used singly and is worthy of clinical trial in this case also.

The mechanisms underlying the synergistic interaction between ICRF-154 and these agents are obscure. ICRF-187 has been reported to enhance free radical formation from doxorubicin without affecting doxorubicin uptake in HL-60 cells (Monti *et al.*, 1990). This may be one of the mechanisms underlying the synergistic interaction of bis(2,6-dioxopiperazine) derivatives and doxorubicin. Although there is still considerable controversy over the mechanism of action of bis(2,6-dioxopiperazine) derivatives (Herman *et al.*, 1982; Sharpe *et al.*, 1970), recent data suggest that topoisomerase-II is involved in their action (Ishida *et al.*, 1991). Amsacrine, doxorubicin, and etoposide, which show supra-additive effects with ICRF-154, are also topoisomerase-II reactive agents (Tewey *et al.*, 1984; Ross *et al.*, 1985). However, bis(2,6-dioxopiperazine) derivatives do not cause formation of a cleavable complex as do the anticancer agents, amsacrine, anthracyclines, and etoposide (Tanabe *et al.*, 1991). We were unable to find any supra-additive effects among amsacrine, doxorubicin, and etoposide (Kano *et al.*, unpublished data), or between ICRF-154 and the topoisomerase-I inhibitor, CPT-11 (Hsiang *et al.*, 1985) against MOLT-3 cells. Whether amsacrine, doxorubicin, and etoposide enhance ICRF-154 cytotoxicity or vice versa is obscure. However, only additive effects were observed between ICRF-154 and doxorubicin in K-562 cells and a human colon carcinoma cell line WiDr (data not shown). The mechanisms underlying these differing interactions in lymphoblastic and other cell lines are not known. Cells of lymphoblastic and myelogenous lines respond to topoisomerases I and II in a very different fashion (Del Bino *et al.*, 1991). The use of available techniques to study DNA topoisomerases may enable the elucidation of the differing cytotoxic mechanisms of topoisomerase inhibitors in combinations against malignant cells of different histological origin.

Our data show that cisplatin had additive effects with ICRF-154 against three lymphoblastic leukaemia cell lines, while it had sub-additive to protective effects against K-562 cells and WiDr (data not shown). Cytosine arabinoside had additive effects with ICRF-154 against all cell lines studied. With respect to the survival of leukaemic mice, the effect of

**Figure 5** Isobolograms of ICRF-154 in combination with other agents in HSB (**a**–**e**), B-ALL (**f**–**j**), and K-562. (**k**–**p**). The agents combined with ICRF-154 are; **a**, **f**, **k**, amsacrine (AMSA); **b**, **g**, **l**, bleomycin (BLM); **m**, cisplatin (CDDP); **c**, **h**, **n**, doxorubicin (DOX), d, i, o, etoposide (VP-16); and **e**, **j**, **p**, methotrexate (MTX).

cisplatin or cytosine arabinoside in combination with ICRF-159 on survival has been reported to be significantly superior to that of either single agent (Kline, 1974).

However, the enhanced activity of a drug combination, whether in animal experiments or in clinical studies, does not require a supra-additive (synergistic) effect. By improved isobologram analysis, additive and even sub-additive effects can show the superiority of drug combinations. Therefore, CPT-11, 5-fluorouracil, mitomycin C, and vincristine, which also showed additive effects with ICRF-154 in our experiment, would be expected to have a stronger cytotoxic effect in combination with ICRF-154 (MST-16) than the single agents in both animal and clinical studies.

Our data show that methotrexate had sub-additive and protective effects with ICRF-154 against all leukaemic cell lines studied. Protection means superiority of the single agents to the combination in terms of effectiveness. In leukaemic mice, the combination of ICRF-159 with methotrexate has been reported to produce an increase in life span only slightly greater than that found when either drug was used separately (Kline, 1974). These findings suggest that the simultaneous administrations of ICRF-154 (MST-16) with methotrexate is unlikely to be advantageous, even though the two drugs have no cross resistance. The mechanisms underlying the antagonistic effects of these combinations are unclear. If ICRF-154 (MST-16) is combined with methotrexate, other suitable schedules should be explored, since the administration schedule (exposure) of drugs may have great influence on the effects of drug combinations (Kano *et al.*, 1988).

In conclusion, we have shown that the effects of ICRF-154 in combination with other anticancer agents are variable. Of the anticancer agents we studied, amsacrine, bleomycin, doxorubicin, and etoposide showed supra-additive effects with ICRF-154 against lymphoblastic leukaemia cell lines.

Combinations of ICRF-154 (MST-16) with these agents should produce a significant clinical response. However, more investigations are necessary to study the effects of ICRF-154 (MST-16) in combination in various cell lines, to ascertain the exact mechanism of this synergism with other agents in lymphoblastic leukaemia cell lines, and to evaluate the toxicity of these combinations. In contrast, methotrexate had an antagonsitic effect with ICRF-154 in all cell lines studied, suggesting that simultaneous administration of these two agents would provide little benefit in the treatment of cancer.

Although there are gaps between *in vitro* studies and clinical trials, these data should provide useful information for the establishment of clinical protocols involving MST-16 ICRF-154 (MST-16).

### References

ADAMS, R.A., FLOWERS, A. & DAVIS, B.J. (1968). Direct implantation and serial transplantation of human acute lymphoblastic leukemia in hamsters. *SB-2. Cancer Res.*, **28**, 1121.

CHOU, T.-C. & TALALAY, M. (1984). Quantitative analysis of dose-effects relationship: the combined effects of multiple drugs on enzyme inhibitors. *Adv. Enzyme Regul.*, **22**, 27.

CREAVEN, P.J., ALLEN, L.M. & ALFORD, D.A. (1975). The bioavailability in man of ICRF-159, a new oral antineoplastic agent. *J. Pharm. Pharmacol.*, **27**, 914.

CREIGHTON, A.M., HELLMANN, K. & WHITECROSS, S. (1969). Antitumor activity in a series of bisdiketopiperazines. *Nature*, **222**, 384.

DEL BINO, G. & DARZYNKIEWICZ, Z. (1991). Camptothecin, teniposide, or 4'-(9-acridinylamino)-3-methanesulfon-m-anisidide, but not mitroxantrone or doxorubicin, induces degradation of nuclear DNA in the S phase of HL-60 cells. *Cancer Res.*, **51**, 1165.

FISHER, V.W., WANG, G.M. & HOBART, N.H. (1986). Mitigation of an anthracycline-induced cardiomyopathy by pretreatment with razoxane: a quantitative morphological assessment. *Vichows Arch. (Cell Pathol)*, **51**, 353.

GRECO, F.A., JOHNSON, D.H. & HAINSWORTH, J.D. (1990). Chronic daily administration of oral etoposode. *Semi. Oncol.*, **8**, 1613.

HELLMAN, K., NEWTON, K.A., WHITMORY, D.W., HANMAN, W.F. & BOND, J.V. (1969). Preliminary clinical assessment of ICRF-159 in acute leukemia and lymphosarcoma. *Br. Med. J.*, **1**, 822.

HERMAN, E.H. & FERRANS, V.J. (1981). Reduction of chronic doxorubicin cardiotoxicity in dogs by pretreatment with (+)-1,2-bis(3,5-dioxopiperazinyl)propane (ICRF-187). *Cancer Res.*, **41**, 3435.

HERMAN, E.H., WITIAK, D.T., HELLMANN, K. & WARAVDEKAR, V.S. (1982). Biological properties of ICRF-159 and related bis(dioxopiperazine) compounds. *Adv. Pharmacol. Chemother.*, **19**, 249.

HSIANG, Y.-H., HERTZBERG, R., HECHT, S. & LIU, L.F. (1985). Camptothecin induces protein linked DNA breaks via mammalian DNA-topoisomerase-I. *J. Biol. Chem.*, **260**, 14873.

ICHIHASHI, T., KIIYO, H., KITAMURA, K. & 6 others (1990). The effects of MST-16 in the treatment of refractory acute leukemia and malignant lymphoma. *Proc. Jap. Hematol. Sco.*, **53**, 113.

ISHIDA, R., MIKI, T., NARITA, T. & 5 others (1991). Inhibition of intracellular topoisomerase II by antitumor bis(2,6-dioxopiperazine) derivatives: mode of cell growth inhibition distinct from that of cleavable complex-forming type inhibitors. *Cancer Res.*, **51**, 4909.

KANO, Y., OHNUMA, T., OKANO, T. & HOLLAND, J.F. (1988). Effects of vincristine in combination with methotrexate and other antitumor agents in human acute lymphoblastic leukemia cells in culture. *Cancer Res.*, **48**, 351.

KANO, Y., SAKAMOTO, S., KASAHARA, T., AKUTSU, M., INOUE, Y. & MIURA, Y. (1991). *In vitro* effects of amsacrine in combination with other anticancer agents. *Leukemia Res.*, **15**, 1059.

KANO, Y., SUZUKI, K., AKUTSU, M. & 5 others (1992). Effects of CPT-11 in combination with other anticancer agents in culture. *Int. J. Cancer*, **50**, 604.

KLINE, I. (1974). Potentially useful combinations of chemotherapy detected in mouse tumor systems. *Cancer Chemother. Rep.*, Part 2, **4**, 33.

KUBONISHI, I., DAIBARA, M., YANO, S. & 7 others (1991). Establishment of a new Epstein-Barr virus nuclear antigen-positive B-cell line, BALL-2, with t(8;14) (q24;q32) chromosome abnormality from B-cell acute lymphoblastic leukemia, L2. *Am. J. Hematol.*, **37**, 179.

LOEWE, S. (1953). The problem of synergism, additivitism and antagonism of combined drug. *Arzneim. Forsch.*, **3**, 285.

LOZZIO, C.B. & LOZZIO, B.B. (1975). Human chronic myelogenous leukemia cell line with positive Philadelphia chromosome. *Blood*, **45**, 321.

MINOWADA, J., OHNUMA, T. & MOORE, G.E. (1972). Rosette-forming human lymphoid cell lines. Establishment and evidence for origins of thymus-derived lymphocytes. *J. Natl Cancer Inst.*, **49**, 891.

MONTI, E. & SINHA, B.K. (1990). Potentiation of doxorubicin cytoxicity by (+)-1.2-bis(3.5-dioxopiperazinyl-1-yl)propane(ICRF-187) in human leukemia HL-60 cells. *Cancer Commu.*, **2**, 145.

MOSMANN, T. (1983). Rapid colormetric assay for cellular growth and survival: application to proliferation and cytotoxicity assay. *J. Immunol. Methods*, **65**, 55.

NARITA, T., YAGUCHI, S., KOMATSU, T. & 4 others (1990). Antitumor activity of MST-16, a novel derivative of bis(2,6dioxopiperazine), in murine tumor models. *Cancer Chemother. Pharmacol.*, **26**, 193.

NARITA, T., YAGUCHI, S., TAKASE, M., INABA, M. & TSUKAGOSHI, T. (1991). Antitumor activities and schedule dependence of orally administrated MST-16, a novel derivative of bis(2,6dioxopiperazine). *Cancer Chemother. Pharmacol.*, **28**, 235.

OHNO, R., YAMADA, K., HIRANO, M. & 11 others (1992). Phase II study: treatment of Non-Hodgkin's lymphoma with an oral antitumor derivative of bis(2,6-dioxopiperazine). *J. Natl Cancer Inst.*, **84**, 435.

ROSS, W., ROWE, T., GLISSON, B., YALOWICH, J. & LIU, L.F. (1985). Role of topoisomerase-II in mediating epipodophyllotoxin-induced DNA cleavage. *Cancer Res.*, **45**, 5872.

SAMPI, K., OGAWA, M., MAEKAWA, T. & 12 others (1985). Phase II study of VP-16 (capsule) on malignant lymphomas. A cooperative study. *Jpn. J. Cancer Chemother.*, **12**, 314.

SHARPE, H.B.A., FIELD, E.O. & HELLMANN, K. (1970). Mode of action of the cytostatic agent 'ICRF-159'. *Nature*, **226**, 524.

STEEL, G.G. & PECKHAM, M.J. (1979). Exploitable mechanisms in combined radiotherapy-chemotherapy: the concept of additivity. *Int. J. Radiat. Oncol.*, **5**, 85.

TANABE, K., IKEGAMI, Y., ISHIDA, R. & ANDOH, T. (1991). Inhibition of topoisomerase II by antitumor agents bis(2,6-dioxopiperazine) derivatives. *Cancer Res.*, **51**, 4903.

TEWEY, K.M., CHEN, G.I., NELSON, E.M. & LIU, L.F. (1984). Intercalative antitumor drugs interfere with the breakage-reunion reaction of mammalian DNA topoisomerase II. *J. Biol. Chem.*, **259**, 9182.

TOMINAGA, N., YOSHIMURA, M., MATSUNASI, T. & 6 others (1990). Effect of MST-16 on hematological neoplasia. *Proc. Jap. Cancer Assoc.*, **49**, 376.

VALERIOTTE, F. & LIN, H. (1975). Synergistic interaction of anticancer agents. A cellular perpective. *Cancer Chemother. Rep.*, **59**, 895.

WADLER, S., GREEN, M.D. & MUGGIA, F.M. (1986). Synergistic activity of doxorubicin and the bisdioxopiperazine (+)-1.2-bis(3,5-dioxopiperazinyl-l-yl)propane(ICRF-187) against the murine sarcoma S180 cell line. *Cancer Res.*, **46**, 1176.

WOODMAN, R.J. (1974). Enhancement of antitumor effectiveness of ICRF-159 (NSC-129943) against early L1210 leukemia by combination with cis-diamminedichloroplatinum (NSC-119875) or daunomycin (NSC-82151). *Cancer Chemother. Rep.*, part 2, **4**, 45.

WOODMAN, R.J., CYSYK, R.L., KLINE, I., GANG, M. & VENDITTI, J.M. (1975). Enhancement of the effectiveness of daunorubicin (NSC-82151) or adriamycin (NSC-123127) against early mouse L-1210 leukemia with ICRF-159 (NSC-129943). *Cancer Chemother. Rep.*, **59**, 689.

YOSHIDA, M., KUSUMOTO, H., TAKAHASHI, I., EMI, Y., KOHNOE, T., SAKAGUCHI, Y., MAEHARA, Y., SUGIMACHI, K. & FUKUDA, S. (1991). Reductive effect on chronic toxicity induced by adriamycin in combination with MST-16. *Proc. Jap. Soc. Cancer Res.*, **29**, 1079.